# Early weight-bearing following surgical fixation of ankle fractures without trans-syndesmotic fixation: safety and early functional outcomes - a systematic review and meta-analysis

**DOI:** 10.1186/s12891-026-09858-y

**Published:** 2026-04-20

**Authors:** Dimitrios Nikos, Nicholas Dominguez, Reuben Chua, Ruurd L. Jaarsma

**Affiliations:** 1 College of Medicine and Public Health, Flinders University, Bedford Park, South Australia Australia; 2 Department of Orthopaedic Surgery, Flinders Medical Centre, Bedford Park, South Australia Australia

**Keywords:** Early weight bearing, Delayed weight bearing, Postoperative rehabilitation, Ankle fracture

## Abstract

**Background:**

Early weight-bearing (EWB) following surgical fixation of malleolar (distal fibula and tibia) fractures in cases without trans-syndesmotic fixation is increasingly advocated, yet clear benefits over delayed weight-bearing (DWB) remain uncertain.

**Methods:**

We systematically searched PubMed, Embase, and Cochrane CENTRAL through April 2025 for randomised and comparative studies evaluating EWB (≤ 3 weeks) versus DWB (≥ 6 weeks) in operatively treated ankle fractures without trans-syndesmotic fixation. Primary outcomes were functional recovery - measured by each trial’s validated ankle-specific score (OMAS or FAOS) at 12 weeks - and overall complication rate, including loss of reduction/malunion/nonunion rates. Return to work/normal daily activities, and all other outcomes were exploratory. Risk of bias was assessed using RoB 2 and ROBINS-I tools.

**Results:**

Eight studies (*n* = 817; 4 RCTs, 4 comparative cohorts) met inclusion criteria. EWB was associated with a statistically significant improvement in functional recovery at 12 weeks (SMD 0.22; 95% CI 0.07–0.38; I² = 0%) without increasing overall complication risk (RR 0.78; 95% CI 0.53–1.14; I² = 0%), although the magnitude of functional improvement remained below minimal clinically important difference (MCID) thresholds. This small statistical difference persisted at 12 months (SMD 0.17; 95% CI 0.02–0.33), remaining below MCID thresholds. No significant differences were found in individual complication subtypes. EWB was also associated with a 10.8 days (MD − 10.82; 95% CI − 17.61 to − 4.04; *P* = 0.002; I² = 35%) earlier return to work/normal daily activities. Subgroup analysis suggested greater functional benefit with CAM boots and when weight-bearing began between postoperative days 10–21, though no significant subgroup interactions were observed.

**Conclusion:**

In surgically treated ankle fractures without trans-syndesmotic fixation, EWB appears safe and is associated with small improvements in early functional recovery and earlier return to activity, without increasing complications. However, the magnitude of functional improvement remained below established MCID thresholds, indicating limited clinical significance and no clear long-term functional superiority over delayed protocols.

**Supplementary Information:**

The online version contains supplementary material available at 10.1186/s12891-026-09858-y.

## Level of evidence

Level 2 (systematic review and meta-analysis of Randomised Controlled Trials and Comparative (Prospective/retrospective studies).

## Introduction

Ankle fractures make up about 10% of all fractures worldwide, with the Swedish Fracture Register reporting an annual incidence of approximately 70 per 100,000 people, resulting in over 56,000 cases over a 10-year period in Sweden [1, 2]. These injuries vary in complexity, from simple isolated malleolar fractures (AO/OTA 44 A) to more unstable bimalleolar and trimalleolar patterns (AO/OTA 44B and 44 C) [3].

The standard surgical approach for unstable fractures is open reduction and internal fixation (ORIF), typically involving the use of plates and screws [4]. Postoperatively, weight-bearing protocols traditionally err on the side of caution, advocating for 6 to 8 weeks of delayed weight-bearing (DWB) to protect the surgical construct, facilitate incision healing and promote fracture healing. This conservative strategy was developed due to concerns of loss of reduction, implant failure and nonunion - particularly in high-risk patients [5]. However, prolonged immobilisation increases the risk of muscle atrophy, joint stiffness, and venous thromboembolism (VTE), delays return to independent function, lengthens hospital stays and increases healthcare utilisation [6, 7].

Early weight-bearing (EWB) is a more recent postoperative approach that involves initiating partial or full weight-bearing within the first three weeks postoperatively, often with the support of a brace or boot - encouraging joint mobility and preserving muscle mass [8]. Conversely, DWB typically restricts any load on the affected limb for 6 to 8 weeks following surgery, often employing immobilisation methods such as a cast or a controlled ankle motion (CAM) boot [9]. Over the past two decades, evidence has emerged suggesting that EWB may be safe and beneficial in selected patients [10–15]. Randomised trials and cohort studies have linked EWB to improved short-term function, faster mobilisation, and no significant rise in complications [16–21].

However, existing evidence remains highly heterogeneous, often encompassing diverse fracture patterns, surgical techniques, and inconsistently applied definitions of weight-bearing protocols. A major confounding factor is the inclusion of syndesmotic injuries, which have distinct biomechanical properties and alter joint mechanics, potentially obscuring the true impact of early mobilisation [22, 23]. Syndesmotic injuries occur in 10 to 23% of all ankle fractures and 20 to 25% of surgical cases, indicating that most operatively managed fractures do not require syndesmotic fixation and represent a substantial population in whom EWB may be safely implemented [24].

To address this gap, our review focused on operatively treated ankle fractures managed without trans-syndesmotic stabilisation, aiming to provide a clinically applicable synthesis of current evidence. To date, no systematic reviews or meta-analyses have exclusively examined EWB in this population. This review assessed the safety and effectiveness of EWB, initiated within 0 to 3 weeks postoperatively, compared to delayed protocols (6 to 8 weeks) in adults undergoing ankle fracture fixation without trans-syndesmotic fixation.

## Methods

### Protocol and reporting guidelines

This systematic review and meta-analysis was conducted in accordance with the Preferred Reporting Items for Systematic Reviews and Meta-Analyses (PRISMA) guidelines [25] and was registered with PROSPERO (CRD420251040060).

### Retrieval strategy

A comprehensive search of PubMed, Embase, and Cochrane CENTRAL was conducted from database inception to April 20, 2025. The strategy combined MeSH terms and free-text keywords covering ankle fractures, operative fixation, and postoperative weight-bearing protocols, including specific fracture patterns, surgical techniques (e.g., ORIF), and loading strategies (e.g., early or delayed weight-bearing). Boolean operators and truncation were used to enhance retrieval. We contacted authors of abstracts and registry-listed trials for unpublished data and included any we received. Reference lists of all included articles and recent reviews were hand-searched for additional studies. The full search strategy is detailed in the Supplementary Material.

### Study selection

**Fig. 1:**
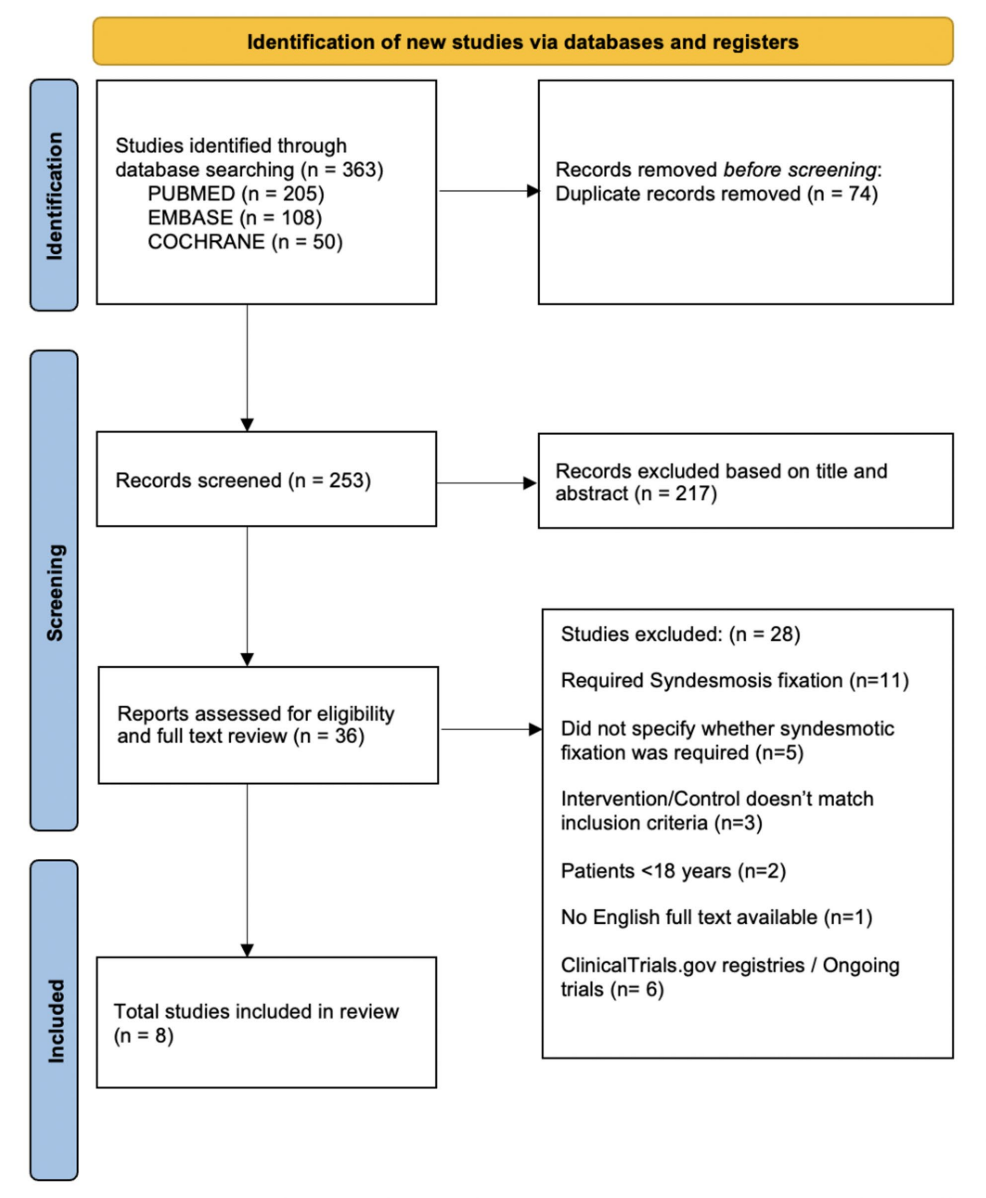
PRISMA flow diagram

All search results were imported into Zotero, and duplicates were removed. Two reviewers (DN, ND) independently screened titles/abstracts and reviewed full texts. Studies involving trans-syndesmotic fixation, or those lacking clear fixation details, were excluded. Discrepancies were resolved by consensus by a third reviewer (RC). The study selection process followed is illustrated in the PRISMA flow diagram (Fig. 1).

### Eligibility criteria

Criteria for inclusion were comparative studies of adults (≥ 18 years) undergoing open reduction and internal fixation for unimalleolar, bimalleolar, or trimalleolar ankle fractures in which the ankle mortise was judged stable after fracture fixation and did not require trans-syndesmotic fixation. Absence of trans-syndesmotic fixation was defined as no implant crossing the distal tibiofibular syndesmosis (e.g., syndesmotic screw or suture-button), irrespective of posterior malleolar fixation. Eligible studies compared EWB (partial or full weight-bearing initiated ≤ 3 weeks postoperatively) with DWB (≥ 6 weeks) and reported functional outcomes, fracture healing, or complications, including loss of reduction. The ≤ 3-week threshold was prespecified a priori to classify early postoperative weight-bearing protocols. This definition represents a pragmatic grouping reflecting commonly reported clinical practice and acknowledges overlap between adjacent regimens, rather than a biologically distinct threshold separating different rehabilitation strategies. Randomised controlled trials, prospective cohorts, and retrospective comparative studies were included. Exclusion criteria were trans-syndesmotic fixation or unclear syndesmotic status, pilon fractures, patients < 18 years, non-operative or non-comparative studies, abstracts or reviews, inaccessible full texts, group sizes < 10, and non-English publications.

### Data extraction

Two reviewers (DN, ND) independently extracted data on study design, participant characteristics, treatment protocols, and outcomes. Most studies reported data at multiple timepoints.

### Data items/outcome measures

Primary outcomes included functional recovery (OMAS or FAOS-ADL at 12 weeks) and complication rates, including loss of reduction, malunion, and nonunion. The 12-week timepoint was prespecified a priori as the most common early functional assessment across trials. Where not explicitly defined, loss of reduction was inferred from follow-up protocols and radiographs, typically obtained at 2, 6, and 12 weeks, using qualitative or threshold-based assessments (e.g., > 2 mm displacement or angular change) (Supplementary Material; Table S1). Exploratory outcomes included functional scores at other relevant timepoints, return to work/normal daily activities, pain and health-related quality of life. Data on immobilisation strategy, fixation construct, fracture pattern (unimalleolar, bimalleolar, or trimalleolar), timing of definitive ORIF, and use of staged management with temporary external fixation were extracted where reported; when not specified, these variables were recorded as not stated. Immobilisation devices (cast, boot, brace, or none), duration of immobilisation, and timing of ankle range-of-motion (ROM) initiation were also extracted, as reduced immobilisation and earlier ROM may independently influence functional recovery. As EWB was frequently implemented alongside removable immobilisation, earlier ROM, and structured rehabilitation protocols (including physiotherapy initiation and progression of loading), the independent effect of axial loading could not always be separated from these co-interventions, and these factors were considered potential confounders in the interpretation of outcomes. Postoperative weight-bearing protocols were classified as partial weight-bearing, weight-bearing as tolerated, or non-weight-bearing based on the authors’ stated definitions; use of protective devices and progression to full weight-bearing were recorded where reported (Table 1). “Weight-bearing as tolerated” was defined according to each study’s protocol and generally referred to progressive loading permitted based on patient tolerance, pain, and clinical guidance, with or without protective immobilisation. Methods used to assess syndesmotic stability and justify omission of trans-syndesmotic fixation - including intra-operative stress testing, medial clear space evaluation, fluoroscopy, or surgeon judgement - were extracted where reported and are summarised in Table 1.

**Table 1:** Baseline Characteristics of Included Studies

Study (Author, Year)	Design	*N* (I/C)	Participant Characteristics & Fracture Type	Surgical Technique	Syndesmosis Assessment	Intervention (Weight-Bearing Strategy & ROM)	Control (Immobilisation & ROM)	Main Outcome(s)	Follow-up (months)	Complications Reported
Simanski, 2006 [26]	Prospective cohort	46 (23/23)	Mean age 55 Weber B & C; uni- or bimalleolar rotational fractures; pilon excluded	ORIF with 1/3 tubular plate and screws	Intra-operative hook, posterior drawer & Frick tests confirming syndesmotic stability	Immediate PWB (10–15 kg) in brace; immediate ROM; progress to WBAT at 3 weeks if radiographs satisfactory	Cast + NWB 6 weeks; ROM restricted; WBAT + ROM after cast removal	Olerud and Molander score, Tegner, RTW, pain intensity	20 ± 11	2 (EWB) vs. 4 (NWB) not significant
Dehghan, 2016 [27]	RCT	110 (56/54)	Mean age 42 Unstable uni-, bi-, trimalleolar fractures; rotational patterns	ORIF with standard lag screw + plate constructs	Intra-op stress test (not specified); unstable cases excluded	WBAT from 2 weeks in CAM boot; daily ankle ROM; wean boot 6–10 weeks	Cast + NWB 2–6 weeks; ROM from 6 weeks; WBAT in boot, wean 6–10 weeks	RTW, Olerud and Molander score, ankle ROM, SF-36	12	No difference; no fixation failures or loss of reduction
Schubert, 2020 [28]	RCT	50 (25/25)	Mean age 44 AO 44B1-3, 44C2; uni-, bi-, trimalleolar; pilon excluded	ORIF (AO principles, plates, and screws)	Intra-op fluoroscopic syndesmotic stress test.	WBAT from 2 weeks in CAM boot; ankle/subtalar ROM from 2 weeks	NWB in CAM boot 6 weeks; ROM allowed; WBAT after 6 weeks	EQ-5D, Olerud and Molander score	6	No fixation failures; 3 DVTs in EWB vs. 1 in NWB; 1 superficial infection (EWB)
Passias, 2020 [29]	Retrospective cohort	95 (38/57)	Mean age 49 Bimalleolar & bimalleolar-equivalent fractures; rotational	ORIF with screws and plates; standard trauma protocol	Intra-op fluoroscopic stress exam; stable mortise required, syndesmotic fixation excluded.	PWB → WBAT from 3 weeks in splint; ROM not specified	NWB in splint until radiographic union (~ 6 weeks); ROM not specified	Radiographic Union, implant failure	12	No difference in implant failure or loosening between groups
Smeeing, 2020 [30]	RCT	115 (78/37)	Mean age 38 Lauge-Hansen SE stage 2–4; rotational fractures	ORIF (surgeon choice - plates, screws, buttress, tension band as indicated)	Intra-operative syndesmosis testing; cases requiring syndesmosis screw excluded	Immediate WBAT (unprotected)/PWB in cast from day 10; ROM after cast removal	NWB (unprotected) with crutches 6 weeks; ROM from 6 weeks	Olerud and Molander score, RTW, SF36	12	No fixation failures or reoperations noted
Park, 2021 [31]	RCT	194 (95/99)	Mean age 43 Unstable uni- and bimalleolar fractures; rotational	ORIF (plates, screws, surgeon discretion)	Intra-op stability assessment; syndesmotic fixation or residual clear-space widening excluded.	WBAT from 2 weeks in removable walking cast, ROM from 2 weeks; cast off at 6 weeks	NWB 0–6 weeks (posterior splint 0–2 weeks, removable splint thereafter); ROM from 2 weeks; splint off at 6 weeks	Olerud and Molander score, RTW	12	3 k-wire backouts (1EWB, 2NWB), no reduction loss
Lee, 2022 [32]	Retrospective matched cohort study	100 (50/50)	Mean age 47 Lateral malleolar fractures (Weber A/B); pilon excluded	ORIF with anatomical locking compression plate + lag screw	Radiographic and intra-operative assessment; cases requiring syndesmotic fixation excluded.	WBAT immediately in air-cast brace with crutches (to tolerance); ankle ROM from POD1; FWB by 4 weeks	Air-cast brace 4 weeks; strict NWB 0–4 weeks; ankle ROM from POD1; WBAT from 4 weeks, FWB by 8 weeks	Radiographic Union, FAOS, RTW	30	No loss of reduction; similar minor complication rates in both groups
Herbosa, 2024 [33]	Prospective case-control	107 (52/55)	Mean age 46 Uni-, bi-, trimalleolar; AO 44 A-C; rotational	ORIF with 1/3 tubular plate ± locking screws; posterior malleolus fixed if needed	Intraoperative stress-view radiographs; cases requiring syndesmotic fixation excluded.	WBAT from 2 weeks in CAM boot; ankle ROM from 2 weeks; crutches/scooter weaned as tolerated; PT during WB	NWB until 6 weeks in CAM boot with crutches/scooter; ankle ROM from 2 weeks; WBAT from 6 weeks, progress to FWB	Olerud-Molander score, SF-36, VAS, RTW	12	No significant difference, wound issues 15.4% (EWB) vs. 9.6% NWB

### Data synthesis and statistical analysis

Meta-analyses were conducted using Review Manager 5.4. A random-effects model was applied in accordance with Cochrane Handbook guidelines [34]. Continuous outcomes were pooled using standardised or mean differences; dichotomous outcomes used risk or odds ratios. Between-study variance was estimated using the REML method, with 95% confidence intervals calculated with Wald-type methods. Heterogeneity was assessed using the I² statistic. Analyses were stratified by design (RCTs vs. observational) to reflect differing bias; mixed-design pooled estimates are presented as sensitivity analyses and interpreted cautiously. Sensitivity analyses (leave-one-out) were also performed. Prespecified subgroup analyses explored effects of immobilisation type (e.g., CAM boot, brace) and timing of weight-bearing initiation (immediate: POD 0–3 vs. delayed-early: POD 10–21). These subgroup analyses were prespecified to examine whether observed effects differed across commonly reported early postoperative rehabilitation strategies and initiation windows; however, they were exploratory in nature and not intended to imply a physiological threshold, establish prescriptive timing, or demonstrate superiority of specific immobilisation methods. Missing data were addressed using standard Cochrane imputation methods [34] to minimise exclusion of eligible studies. The Smeeing 2020 trial [30] had two EWB arms (protected, unprotected) sharing a single unprotected NWB control. For the main analysis, we merged EWB arms per Cochrane guidelines; for subgroups, we split the control to avoid double-counting. Functional SMDs were back-translated to OMAS or FAOS–ADL points and benchmarked against published minimal clinically important differences (MCID) [35, 36]. OMAS MCIDs ranged from 10.5 to 15.0 at 3–6 months and 7.5–11.4 at 6–12 months, while FAOS–ADL MCIDs ranged from 8.3 to 10.3. The review was hypothesis-driven with prespecified outcomes; no multiplicity adjustment was applied.

### Risk of bias analysis

The potential for bias in each included study was assessed using the Cochrane Risk of Bias 2 (RoB-2) tool for randomised trials and the ROBINS-I tool for non-randomised studies [37, 38]. Publication bias was examined via funnel plot analysis of primary outcomes (See Supplementary Material).

### Assessment of quality of evidence

Certainty of evidence was rated with Grading of Recommendations, Assessment, Development, and Evaluation (GRADE) framework for each outcome, considering study design, risk of bias, consistency, precision, and clinical relevance [39].

## Results

### Search results

A total of 363 records were retrieved from database searches. After duplicate removal and screening, 36 full-text articles were reviewed in detail. Based on the inclusion and exclusion criteria, 28 articles were excluded - primarily due to inclusion of trans-syndesmotic fixation. Eight studies (4 RCTs and 4 comparative cohorts) were included in the final meta-analysis. The full selection process is illustrated in the PRISMA flow diagram (Fig. 1).

### Overview of included studies

Table 1 summarises the characteristics of the eight studies included in this meta-analysis, comprising 817 patients (417 EWB, 400 DWB). Study sample sizes ranged from 46 to 194 participants, with mean ages between 38 and 55 years. Included injuries comprised unimalleolar, bimalleolar, and trimalleolar ankle fractures, predominantly resulting from rotational mechanisms. Where reported, fractures were classified using the OTA/AO, Weber, and Lauge-Hansen systems, with most injuries falling within the OTA/AO 44 A-44 C spectrum. Supination-external rotation injuries corresponding to Weber B (AO 44-B) fractures predominated, accounting for approximately 86% (699/817) of cases. Weber C (AO 44-C) fractures comprised approximately 10% (80/817) and were more frequently associated with trimalleolar patterns and posterior malleolar involvement, while Weber A (AO 44-A) fractures accounted for the remaining 4% (38/817). All patients underwent ORIF, and no study included trans-syndesmotic fixation; absence of trans-syndesmotic fixation reflected confirmation of syndesmotic stability after fracture reduction rather than systematic exclusion of syndesmotic injury, assessed intra-operatively and/or radiographically, as detailed in Table 1. Reporting of fracture subtypes, posterior malleolar involvement, and fixation strategy was inconsistent across studies, limiting fracture-specific and construct-specific subgroup analyses. Open fractures, pilon fractures and Maisonneuve injuries were consistently excluded. Definitions of EWB varied across studies, including quantified partial weight-bearing protocols (e.g., 10–15 kg) and weight-bearing as tolerated without load specification (Table 1). Postoperative immobilisation and ROM protocols also varied across studies.

### Risk of bias evaluation

All four RCTs had some concerns, mainly from intervention deviations and selective reporting. Non-randomised studies showed moderate risk due to confounding and lack of blinding, with Herbosa et al. [33] rated as serious risk. Overall, methodological quality was variable, with all RCTs demonstrating some concerns and non-randomised studies showing moderate to serious risk of bias, primarily due to confounding and lack of blinding.

### Outcomes

#### Functional recovery outcomes after ankle fracture surgery

Seven studies were included in the analysis of functional outcomes, using validated patient-reported scores, primarily OMAS and FAOS. OMAS was the most commonly reported score, used in six studies [26–28, 30, 31, 33]. Simanski et al. [26] additionally reported outcomes using the Tegner Activity Scale, while Lee et al. [32] assessed function using the FAOS–Activities of Daily Living (ADL) subscale exclusively. Passias et al. [29] did not assess functional outcomes, focusing instead on radiographic healing and complications. OMAS and FAOS-ADL scores were pooled for meta-analysis using standardised mean differences, as both are validated, ankle-specific measures of function. SF-36 and EQ-5D were analysed separately to reduce heterogeneity and maintain clarity in HRQoL outcomes.

#### Primary outcomes

##### Functional recovery at 12 weeks post-surgery

**Fig. 2:**
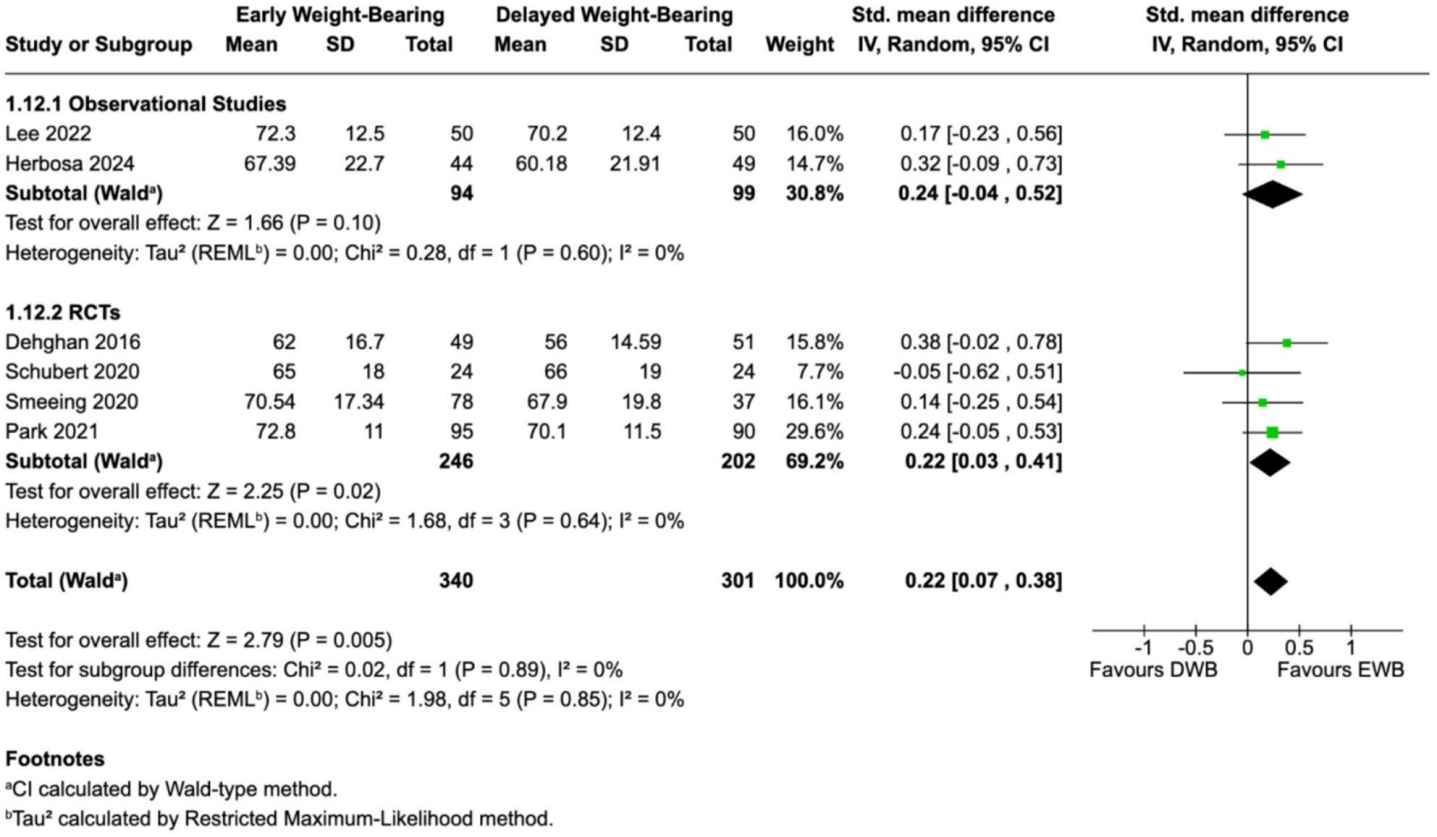
Functional Recovery at 12 weeks post-surgery

Six studies [27, 28, 30–33] reported 12-week functional outcomes. Observational studies showed a small, non-significant effect (SMD 0.24; 95% CI − 0.04 to 0.52), while RCTs demonstrated a significant benefit (SMD 0.22; 95% CI 0.03 to 0.41). The pooled analysis demonstrated a statistically significant advantage with EWB (SMD 0.22; 95% CI 0.07–0.38; I² = 0%), translating to ≈ 3.6 OMAS or ≈ 2.7 FAOS points - both below established MCID thresholds (OMAS 10.5–15.0; FAOS-ADL 8.3–10.3). Despite statistical significance, these values indicate only a small functional advantage for EWB (Fig. 2).

#### Subgroup analyses & sensitivity analysis at 12 weeks post-surgery

Exploratory subgroup analyses suggested somewhat larger early functional effects in studies using removable CAM boot protocols (SMD 0.27; 95% CI 0.02–0.52); however, subgroup interactions were not statistically significant (*P* = 0.91), and these findings should be interpreted cautiously. EWB initiated between POD 10–21 was associated with improved functional outcomes (SMD 0.23; 95% CI 0.05–0.41), whereas initiation on POD 0–3 was not; however, no significant interaction was observed between timing subgroups (*P* = 0.79), and these findings remain exploratory. The overall pooled effect at 12 weeks remained statistically significant (SMD 0.22; 95% CI 0.06–0.38; I² = 0%). Sensitivity analysis excluding the immediate weight-bearing protocol [32] did not materially alter the pooled effect or heterogeneity (see Supplementary Appendix).

#### Complications and safety assessment

**Fig. 3:**
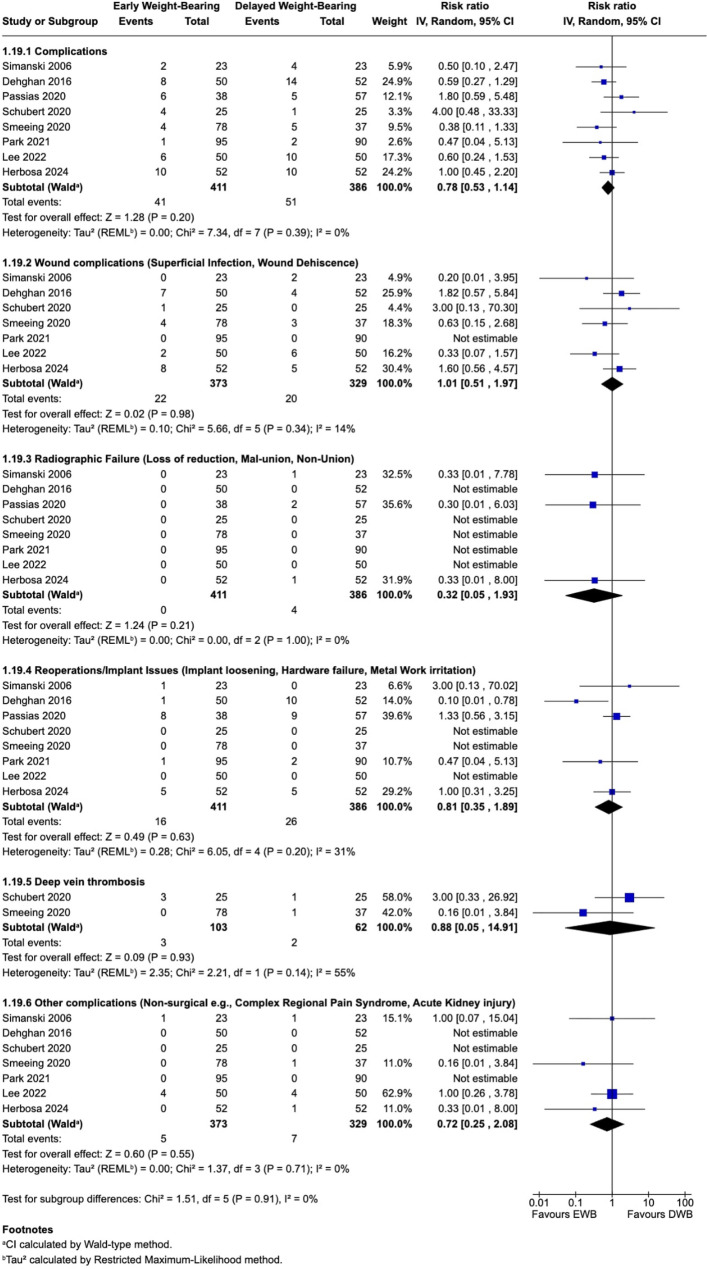
Postoperative Complications with subgroups

All eight included studies [26–33] reported postoperative complications including loss of reduction, malunion, or nonunion (Fig. 3, Table 2). Wound-related complications were the most frequently reported [26–28, 30–33]. Notably, no cases of loss of reduction, malunion, or nonunion were reported in EWB patients (0/417; 0.0%), compared to four events among DWB patients (4/400; 1.0%) across all eight studies. In the pooled analysis, EWB was not associated with increased overall complication risk compared with DWB (RR 0.78; 95% CI 0.53–1.14; I² = 0%). Design-stratified results were consistent: observational studies showed no difference (RR 0.91; 95% CI 0.55–1.50), and RCTs suggested fewer complications with EWB (RR 0.62; 95% CI 0.34–1.13). (See Supplementary Appendix). Meta-analyses of complication subtypes demonstrated no statistically significant differences between EWB and DWB (Fig. 3 and Table 2).

**Table 2:** Postoperative Complications - Breakdown of Complication Types

Complication Type	Early WB — Events (*n*)	Delayed WB — Events (*n*)	Total Events (*n*)	Pooled RR (Random-effects 95% CI)
Wound complications (Superficial infection, Wound dehiscence)	22	20	42	1.01 [0.51, 1.97]
Radiographic Failure (Loss of reduction/Nonunion/Malunion)	0	4	4	0.32 [0.05, 1.93]
Re-operations/implant issues (Implant loosening, hardware failure, metal work irritation)	16	26	42	0.81 [0.35, 1.89]
Deep Vein Thrombosis	3	2	5	0.88 [0.05, 14.91]
Other complications (Non-surgical e.g., Complex regional pain syndrome, Acute kidney injury)	5	7	12	0.72 [0.25, 2.08]

Events are counted individually; patients may have experienced more than one complication

### Exploratory outcomes

#### Functional recovery at other specified timepoints

Five studies [27, 28, 30, 31, 33] reported functional outcomes at 6 weeks. RCTs favoured EWB (SMD = 0.53; 95% CI, 0.31–0.74; I² = 19%), as did the single observational study (SMD = 0.78; 95% CI, 0.39–1.18), with no significant difference between designs (*P* = 0.27). The pooled effect at 6 weeks (SMD 0.58; 95% CI 0.38–0.78; I² = 24%) equates to ≈ 9.3 OMAS points in favor of EWB (95% CI 6.1–12.4).

At 6 months postoperatively, four studies [27, 28, 30, 33] showed a small, non-significant benefit for EWB over DWB (SMD = 0.17; 95% CI, − 0.05–0.40; I² = 0%), equating to ≈ 3.1 OMAS points (95% CI, − 0.9 to 7.4), sub-MCID, though still favouring EWB.

**Fig. 4:**
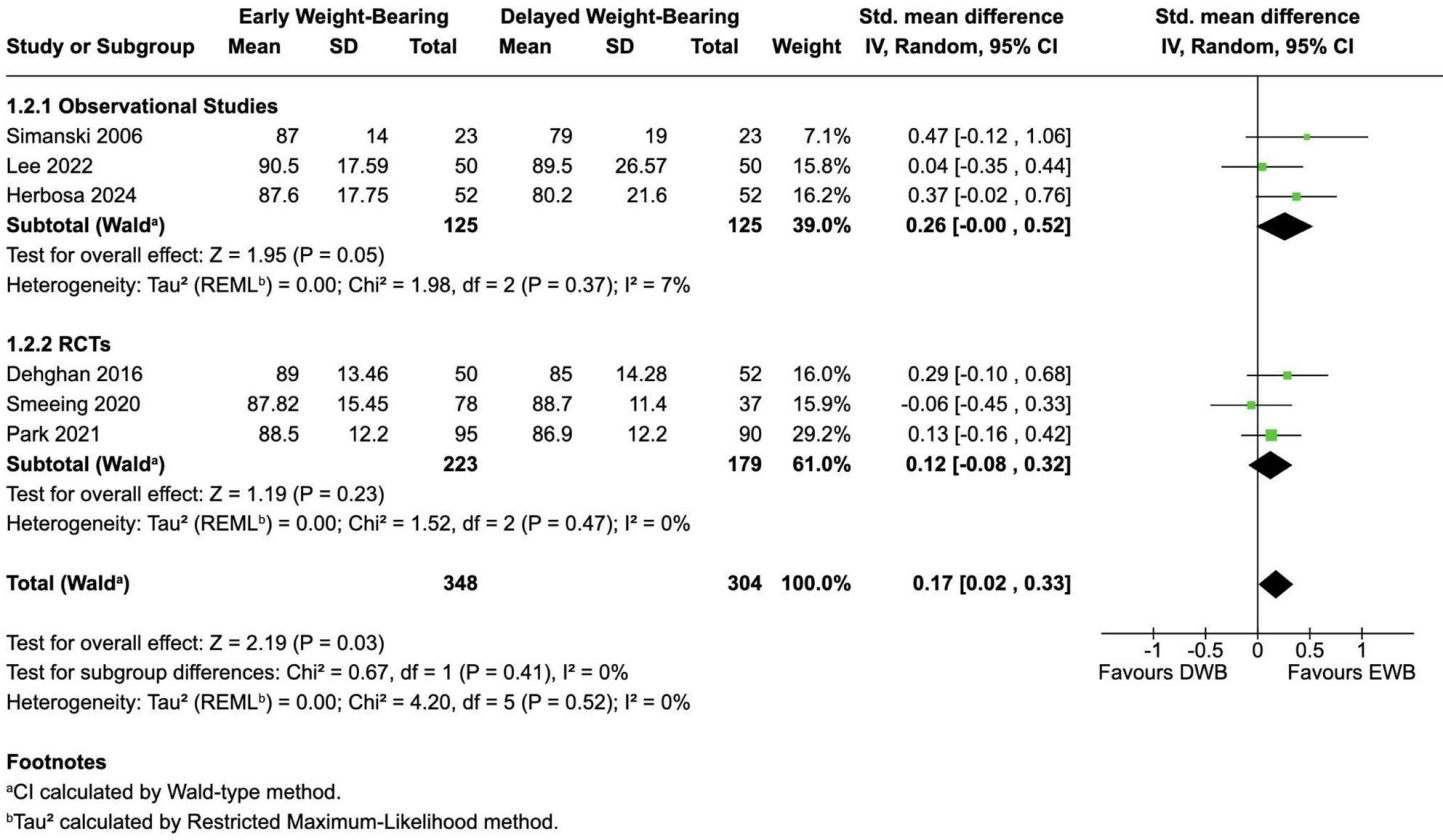
Functional Recovery at 12 months post-surgery

Six studies [26, 27, 30–33] reported functional outcomes at 12 months. Observational studies suggested a benefit of EWB (SMD 0.26; 95% CI − 0.00 to 0.52), whereas RCTs indicated no clear difference (SMD 0.12; 95% CI − 0.08 to 0.32). When pooled, the effect size was modest (SMD 0.17; 95% CI 0.02–0.33; I² = 0%), translating to ≈ 2.8 OMAS (95% CI 0.3–5.4) and ≈ 3.8 FAOS–ADL points (95% CI 0.5–7.4), both below published MCIDs but consistently in favour of EWB (Fig. 4).

#### Return to work/normal daily activities after ankle fracture surgery

Five studies [26, 27, 30–32] assessed return to work or daily activities, generally defined as resuming usual occupational or household tasks, excluding sport or high-impact activity. The two observational studies included indicated a significant reduction in the EWB group of 14.6 days (95% CI − 25.8 to − 3.4; I² = 0%), while RCTs showed a smaller, non-significant effect (MD − 8.9 days; 95% CI − 19.6 to 1.7; I² = 64%). Pooled sensitivity analysis indicated a modest overall benefit (MD − 10.8 days; 95% CI − 17.6 to − 4.0; I² = 35%).

#### Pain scores

Pain was inconsistently reported across studies. Neither group found an improvement in pain scores over the other.

#### Health-Related Quality of Life (HRQoL)

Five studies [27, 28, 30, 32, 33] assessed HRQoL using SF-36 (Physical Component), EQ-5D, or FAOS-QOL at 12 weeks. Pooled analysis showed no significant difference between EWB and DWB (SMD 0.18; 95% CI − 0.00 to 0.37; I² = 0%). Observational and RCT data suggested similar modest effects.

### GRADE / SoF summary / quality of evidence assessment

Certainty of evidence was low to moderate overall: moderate for both primary outcomes and low for most exploratory outcomes (Table 3).

**Table 3:** Summary of Findings: Early Weight-Bearing Following Surgical Fixation of Ankle Fractures Without Trans-Syndesmotic Fixation: Safety and Early Functional Outcomes - A Systematic Review and Meta-Analysis

Early Weight-Bearing Following Surgical Fixation of Ankle Fractures Without Trans-Syndesmotic Fixation: Safety and Early Functional Outcomes - A Systematic Review and Meta-Analysis					
Population: Patients with ankle fractures that have undergone surgery without trans-syndesmotic fixation Setting: Tertiary medical centre Intervention: Weight-bearing within 3 weeks postoperatively Comparison: Weight-bearing from 6 weeks or more postoperatively					

Outcome	No. of Participants (Studies)	Relative Effect (95% CI)	Absolute Effect (95% CI)	Certainty of Evidence (GRADE)	Interpretation
Pooled Functional Outcomes at 12 weeks (Primary)	655 (6 studies)	SMD 0.22 (0.07–0.38)	3.6 OMAS 2.7 FAOS-ADL	**Moderate**	Small improvement in function, favouring EWB
Postoperative Complication Rate (Primary)	798 (8 studies)	RR 0.78 (0.53–1.14)	29 fewer per 1,000 (From 57 fewer to 16 more)	**Moderate**	No difference in overall complication rates. Meta-analyses of complication subtypes demonstrated no statistically significant differences between EWB and DWB
			No of cases of loss of reduction or malunion / nonunion: EWB: 0 DWB: 4		
Pooled Functional Outcomes at 6 weeks (Exploratory)	565 (5 studies)	0.58 (0.38–0.78)	9.3 OMAS	**Moderate**	Moderate improvement in function, favouring EWB (not interpreted as definitive treatment effect)
Pooled Functional Outcomes at 6 months (Exploratory)	320 (4 studies)	0.17 (–0.05 to 0.40)	3.1 OMAS	**Low**	Not statistically significant
Pooled Functional Outcomes at 12 months (Exploratory)	652 (6 studies)	0.17 (0.02–0.33)	2.8 OMAS 3.8 FAOS-ADL	**Low**	Small improvement in function, favouring EWB
Return to work / Normal daily activities (Exploratory)	500 (5 studies)	-	MD − 10.8 days (–17.6 to -4.0)	**Low**	Trend to earlier return to work

## Discussion

This systematic review and meta-analysis evaluated whether EWB - defined as partial or full weight-bearing initiated three weeks or earlier postoperatively - offers functional or safety advantages over DWB following ankle fracture fixation without trans-syndesmotic stabilisation. Eight studies (817 patients) were included; six contributed to the pooled analysis of the primary functional outcome at 12 weeks, which showed a consistent but small functional advantage for EWB, with no increase in overall complication rates.

The analysis focused on operatively treated ankle fractures managed without trans-syndesmotic fixation, thereby reducing confounding from syndesmotic implant constructs and their associated postoperative weight-bearing restrictions. Accordingly, these findings apply to operatively treated ankle fractures in which, after fracture fixation (including posterior malleolar fixation where performed), the mortise was judged sufficiently stable that trans-syndesmotic fixation was not required, consistent with prior comparative evidence [33]. Absence of trans-syndesmotic fixation does not necessarily imply absence of syndesmotic injury; rather, these findings apply to cases in which the ankle mortise was judged sufficiently stable after fracture fixation such that additional syndesmotic stabilisation was not required, noting that emerging techniques such as needle arthroscopy may improve detection of subtle or occult syndesmotic instability beyond conventional intraoperative assessment [40, 41]. Accordingly, the results are most applicable to predominantly Weber B (AO/OTA 44B) fractures with stable fixation, as reflected in the included studies (Table 1) and should not be extrapolated to all ankle fracture patterns. While prior systematic reviews [10, 14, 15] have reported similar safety outcomes with EWB, most pooled fractures with and without syndesmotic fixation; isolating syndesmosis-stable fractures refines existing evidence and provides a focused synthesis for clinicians managing these cases.

EWB is associated with modest improvements in early functional recovery without compromising long-term outcomes. At 12 weeks - the primary functional timepoint - EWB demonstrated a statistically significant advantage; however, the magnitude of this difference remained below established MCID thresholds [35, 36], suggesting the observed effect is unlikely to represent a clinically meaningful difference and reflects a short-term acceleration of recovery rather than superiority. At 6 weeks, higher EWB scores likely reflect protocol-imposed restriction in the delayed group, suggesting earlier independence rather than definitive treatment efficacy. By 6 months, differences had largely diminished, consistent with catch-up in the delayed weight-bearing cohort, and at 12 months outcomes remained comparable. These findings align with prior prospective studies demonstrating early divergence with subsequent convergence over time [33]. RCT data similarly show no meaningful long-term functional advantage of EWB, with early observational gains likely influenced by confounding factors such as rehabilitation protocols and immobilisation strategies.

In terms of safety, all eight included studies reported postoperative complications. Design-stratified analysis showed consistent findings across both observational studies and RCTs. In the pooled analysis, EWB was not associated with an increased risk of postoperative complications compared with DWB and, in fact, suggested a relative reduction of about 22%. These findings are consistent with previous reviews and reinforce that early loading does not increase complication risk [27, 42, 43]. Superficial wound problems were the most frequently reported events, all managed conservatively. Although earlier studies [44] raised concerns about higher wound risks with early mobilisation, several recent RCTs have not supported this, and our analysis likewise found no excess wound-related complications. Although EWB encompassed a spectrum from protected partial loading to weight-bearing as tolerated, no increase in mechanical complications was observed across protocols, and sensitivity analysis confirmed that exclusion of the immediate weight-bearing cohorts did not materially alter the pooled effect. Additionally, EWB demonstrated comparable union rates and no increased risk of malunion, displacement, or fixation failure when compared to DWB (EWB 0/417 (0.0%) vs. DWB 4/400 (1.0%)) [26, 28, 29, 32, 45, 46]. Given the very low event rate, these findings are best interpreted as evidence of non-inferiority of early loading rather than definitive superiority, with apparent differences likely influenced by confounding by indication, protective rehabilitation co-interventions (e.g., CAM boots), and event-rate imprecision. Subgroup analyses revealed no significant differences between EWB and DWB in wound complications, radiographic/mechanical failure, reoperations, or thromboembolic events.

Exploratory subgroup analyses suggested somewhat larger early functional effects in studies using removable CAM boot protocols, with smaller, non-significant effects observed with braces and no clear benefit with casts, splints, or unprotected loading; however, subgroup interactions were not statistically significant, and these findings should be interpreted cautiously. This is broadly consistent with Zhou et al. [47], although differences in rehabilitation protocols limit direct comparison. With respect to timing, EWB initiated between POD 10–21 was associated with improved functional outcomes, whereas immediate unprotected loading (POD 0–3) was not; however, no significant interaction was observed, and these findings remain exploratory. As EWB was frequently implemented alongside broader rehabilitation strategies - including removable immobilisation, earlier ROM, and structured physiotherapy - these factors represent important potential confounders, and observed functional advantages may partly reflect rehabilitation effects rather than the independent effect of axial loading alone, as noted by Dehghan et al. [27]. Overall, these findings should be considered hypothesis-generating rather than practice-directing, and likely reflect differences in rehabilitation strategy rather than a direct effect of weight-bearing alone.

Beyond function and safety, EWB accelerated return to work and independence. EWB was associated with an approximately 11-day earlier return to work/normal activities, representing a clinically meaningful functional advantage during early recovery. The Smeeing et al. [30] trial was an outlier, reporting return at 34 days in the unprotected EWB group, likely due to a younger cohort, intensive rehab, and broad self-reported definitions including partial duties. Sensitivity analysis excluding this study did not materially alter pooled estimates. Earlier mobilisation may additionally reduce rehabilitation costs [48], improve confidence, and lessen care needs, though supporting evidence remains inconsistent [49].

Most included cohorts comprised Weber B (AO/OTA 44-B) supination-external rotation injuries (699/817; ≈86%), with a mean patient age of approximately 46 years. Accordingly, these findings are most applicable to relatively younger adults with predominantly Weber B rotational fracture patterns and should be extrapolated cautiously to Weber C injuries, more complex bimalleolar or trimalleolar fractures, and geriatric or osteoporotic populations; in these higher-risk or more complex scenarios, postoperative loading should be individualised based on fracture stability, fixation quality, patient age, bone quality, and comorbidities [27, 33]. Across included studies, EWB was implemented following restoration of a congruent and stable ankle mortise without trans-syndesmotic fixation, typically alongside removable immobilisation and initiation in the early postoperative period (approximately POD 10–21), with progression tailored to fixation stability, soft-tissue condition, and patient factors [50]. Future studies should standardise reporting of fracture type, fixation strategy, and surgical intent to improve comparability and enable more robust subgroup analyses.

Interpretation of pooled functional outcomes against established MCID thresholds for OMAS and FAOS represents a methodological strength of this review and addresses a limitation of prior meta-analyses in this field [51], allowing statistically significant effects to be distinguished from those of limited clinical relevance. Key limitations included inconsistent reporting of immobilisation strategies, fixation constructs, fracture patterns, and timing of definitive fixation, which precluded robust subgroup analyses. In addition, EWB was frequently implemented within broader rehabilitation protocols, representing an important potential confounder and making it difficult to isolate the independent contribution of axial loading from these co-interventions. The ≤ 3-week definition of EWB represents a pragmatic categorisation rather than a biologically distinct threshold. Within this grouping, protocols ranged from immediate unprotected loading to delayed-early weight-bearing closer to three weeks, which likely represent different biomechanical and rehabilitation strategies. This heterogeneity may dilute true treatment effects when pooled and should be considered when interpreting the findings. While posterior malleolar fixation is commonly performed to restore articular congruity and may contribute to syndesmotic stability via the posterior inferior tibiofibular ligament (PITFL), it is conceptually and functionally distinct from trans-syndesmotic fixation involving an implant crossing the distal tibiofibular syndesmosis [52]. Accordingly, posterior malleolar fixation was not considered equivalent to syndesmotic screw or suture-button fixation, and studies were excluded only when a separate trans-syndesmotic stabilisation device was used [53]. In addition to PITFL-related posterior malleolar fragments, avulsion-type injuries of the syndesmotic complex - particularly anterior inferior tibiofibular ligament (AITFL) avulsion fractures (Wagstaffe-Le Fort lesions) - are increasingly recognised as markers of syndesmotic disruption. Contemporary series suggest these injuries are more common than previously appreciated, with reported prevalence approaching 20% in ankle fracture cohorts and broader avulsion patterns involving the AITFL and PITFL insertions occurring in over one-quarter of adult ankle fractures [54, 55]. These injuries may influence syndesmotic alignment and fibular reduction, and instability may therefore persist despite the absence of trans-syndesmotic fixation. However, reporting of posterior malleolar fixation, its intended stabilising role, and associated syndesmotic avulsion patterns was inconsistent across included studies, limiting fracture-level classification and contributing to clinical heterogeneity.

Injury-to-ORIF interval and use of staged management were also inconsistently reported, limiting adjustment for injury severity and soft-tissue status. Notably, most included studies either explicitly excluded staged fixation or did not report its use, suggesting that the findings primarily reflect single-stage definitive ORIF in patients with acceptable soft-tissue conditions. Excluding studies with trans-syndesmotic fixation [13, 17, 19–21, 43, 44, 56–68] also limited sample size and generalisability but was necessary to isolate EWB effects in fractures without syndesmotic involvement. Risk of bias was present across included studies, with all RCTs rated as having some concerns and non-randomised studies demonstrating moderate to serious risk, particularly due to confounding and lack of blinding. These limitations contributed to moderate GRADE certainty for primary outcomes and should be considered when interpreting the findings.

Historically, early loading post-fixation was discouraged due to fears of displacement or failure [46, 53]. However, consistent with prior biomechanical and clinical studies, our findings confirm EWB does not increase these risks when used appropriately [23, 46, 69]. These findings support the safety of EWB following fixation of ankle fractures that do not require trans-syndesmotic stabilisation, extending prior meta-analyses that either pooled mixed, heterogeneous fracture patterns or focused exclusively on syndesmotic injuries, where biomechanical variability or restricted populations limited the specificity and generalisability of their conclusions [10, 14, 15, 42, 51, 69–72].

## Conclusion

Early weight-bearing was not associated with increased complications when fixation restored a stable ankle mortise without trans-syndesmotic stabilisation, though applicability may vary with patient factors such as comorbidity burden, bone quality, and metabolic risk. It was associated with small improvements in early functional recovery and earlier return to work; however, these gains remained below established MCID thresholds at both 12 weeks and 12 months, indicating limited clinical significance and no clear long-term functional advantage over delayed protocols. Findings are most applicable to predominantly Weber B fracture patterns in relatively younger adults, with cautious extrapolation required for more complex injuries.

## Supplementary Information

Supplementary Material 1: Supplementary Figure 1. Risk of Bias Assessment for Randomized Controlled Trials Using the Cochrane RoB 2 Tool. Supplementary Figure 2. Risk of Bias Assessment for Non-Randomised Studies Using the ROBINS-I Tool. Supplementary Figure 3. Funnel plot evaluating potential publication bias for studies reporting 12-week functional outcomes. Supplementary Figure 4. Subgroup Analysis at 12 Weeks - Immobilisation Device. Supplementary Figure 5. Subgroup Analysis at 12 Weeks - POD Weightbearing initiation. Supplementary Figure 6. Functional Recovery at 6 Weeks Post-Surgery. Supplementary Figure 7. Functional Recovery at 6 Months Post-Surgery. Supplementary Figure 8. Return to Work/Normal daily Activities After Ankle Fracture Surgery. Supplementary Figure 9. Health-Related Quality of Life (HRQoL). Supplementary Figure 10. Post-operative Complications (design stratified analysis).

## Data Availability

All data used in this systematic review were derived from previously published studies. Additional data are available from the corresponding author upon reasonable request.

## Abbreviations

AO: Arbeitsgemeinschaft für Osteosynthesefragen
CAM: Controlled ankle motion
EWB: Early weight-bearing
FAOS: Foot and Ankle Outcome Score
I/C: Intervention/control
NWB: Non-weight-bearing
OMAS: Olerud-Molander Ankle Score
ORIF: Open reduction and internal fixation
PWB: Partial weight-bearing
RCT: Randomised controlled trial
ROM: Range of motion
RTW: Return to work
SE: Supination external rotation
SF-36: 36-Item Short Form Health Survey
VAS: Visual analogue scale
XR: Radiograph
