# Supplementary material for: Early weight-bearing following surgical fixation of ankle fractures without trans-syndesmotic fixation: safety and early functional outcomes - a systematic review and meta-analysis
Source: BMC Musculoskelet Disord. 2026 Apr 20;27:468. doi: 10.1186/s12891-026-09858-y (PMC13224453; doi:10.1186/s12891-026-09858-y)
Supplement: Supplementary file 1 — Supplementary Material 1: Supplementary Figure 1. Risk of Bias Assessment for Randomized Controlled Trials Using the Cochrane RoB 2 Tool. Supplementary Figure 2. Risk of Bias Assessment for Non-Randomised Studies Using the ROBINS-I Tool. Supplementary Figure 3. Funnel plot evaluating potential publication bias for studies reporting 12-week functional outcomes. Supplementary Figure 4. Subgroup Analysis at 12 Weeks - Immobilisation Device. Supplementary Figure 5. Subgroup Analysis at 12 Weeks - POD Weightbearing initiation. Supplementary Figure 6. Functional Recovery at 6 Weeks Post-Surgery. Supplementary Figure 7. Functional Recovery at 6 Months Post-Surgery. Supplementary Figure 8. Return to Work/Normal daily Activities After Ankle Fracture Surgery. Supplementary Figure 9. Health-Related Quality of Life (HRQoL). Supplementary Figure 10. Post-operative Complications (design stratified analysis). [file 12891_2026_9858_MOESM1_ESM.docx]

**SUPPLEMENTARY MATERIAL**

**Full search string**

Pubmed/Cochrane search

("ankle fracture*" OR "fractured ankle" OR "malleolar fracture" OR "lateral malleolar fracture" OR "medial malleolar fracture" OR "posterior malleolar fracture" OR unimalleolar OR bimalleolar OR trimalleolar OR "distal fibula fracture" OR "distal tibia fracture" OR "ankle injury with fracture" OR weber*) AND ( fixation OR "operative treatment" OR "open reduction internal fixation" OR ORIF) AND ("weight-bearing" OR "early weight-bear*" OR "immediate weight-bear*" OR "early mobilisation" OR "early mobilization" OR "functional weight-bear*" OR "unprotected weight-bear*") AND ("non-weight-bear*" OR "nonweightbear*" OR "delayed weight-bear*" OR "prolonged non-weight-bear*" OR NWB OR immobilization OR immobilisation)

Embase search

(ankle fracture/ or ankle fracture* or fractured ankle or malleolar fracture* or lateral malleolar fracture* or medial malleolar fracture* or posterior malleolar fracture* or unimalleolar or bimalleolar or trimalleolar or distal fibula fracture* or distal tibia fracture* or ankle injur* with fracture* or weber*)

(surgical fixation or operative treatment or open reduction internal fixation or ORIF or "open reduction (procedure)"/)

(weight-bearing or early weight-bear* or immediate weight-bear* or early mobili?ation or functional weight-bear* or unprotected weight-bear*)

(non-weight-bear* or nonweightbear* or non-weight bear or delayed weight-bear* or prolonged non-weight-bear* or NWB or immobili?ation)

**Risk of Bias Assessment**

### **
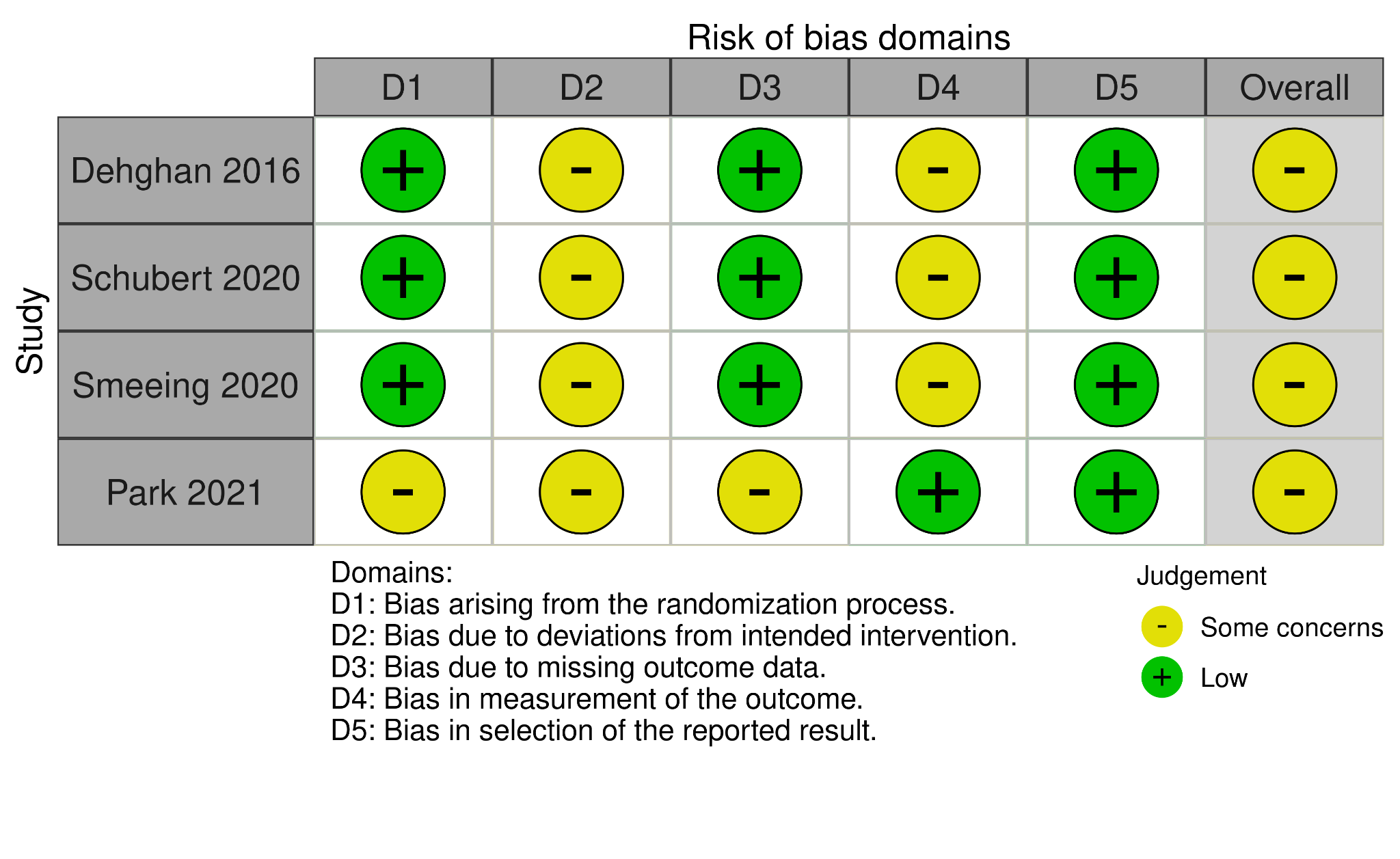
**

**Supplementary Figure 1. Risk of Bias Assessment for Randomized Controlled Trials Using the Cochrane RoB 2 Tool**

**
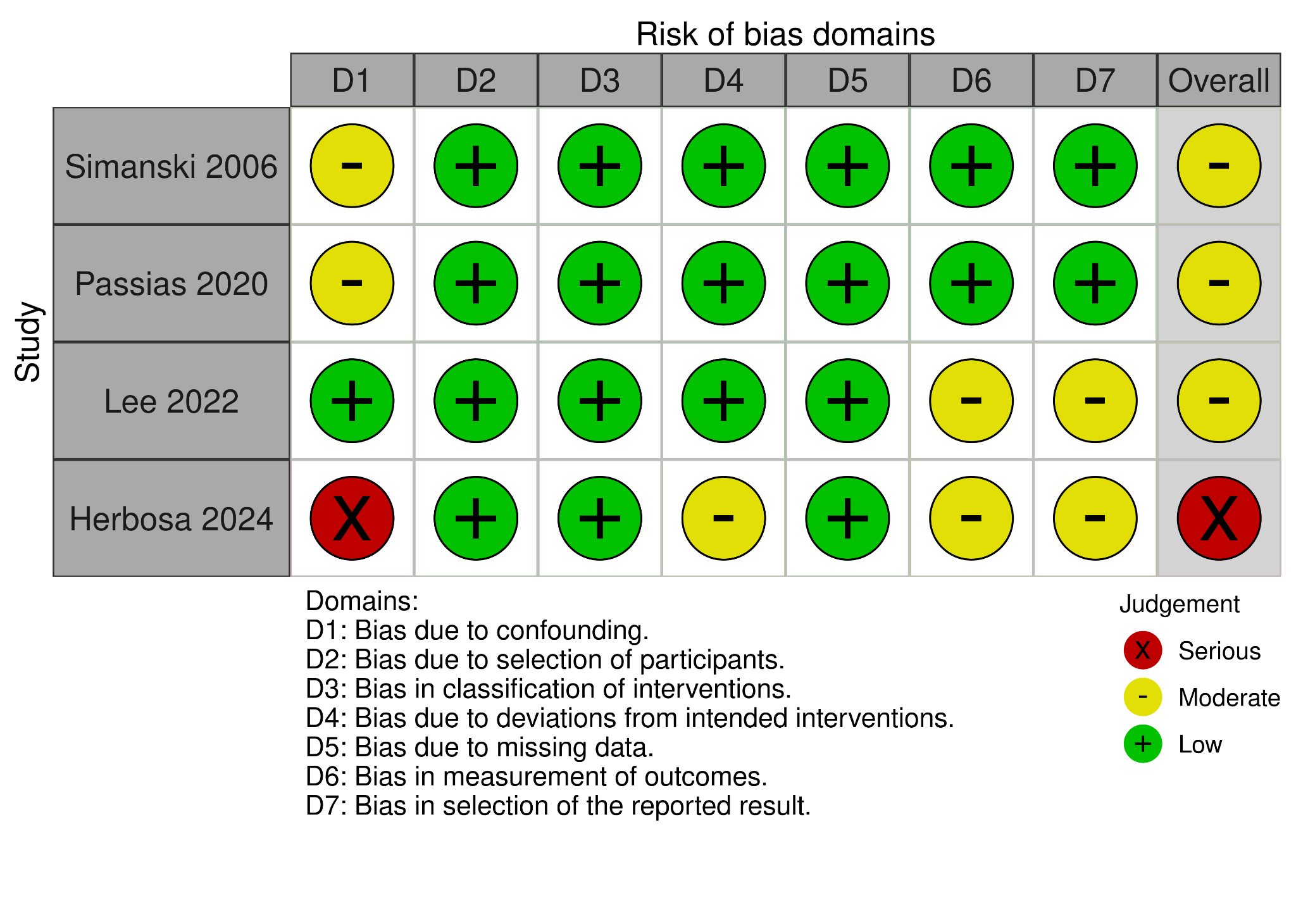
**

**Supplementary Figure 2. Risk of Bias Assessment for Non-Randomized Studies Using the ROBINS-I Tool**


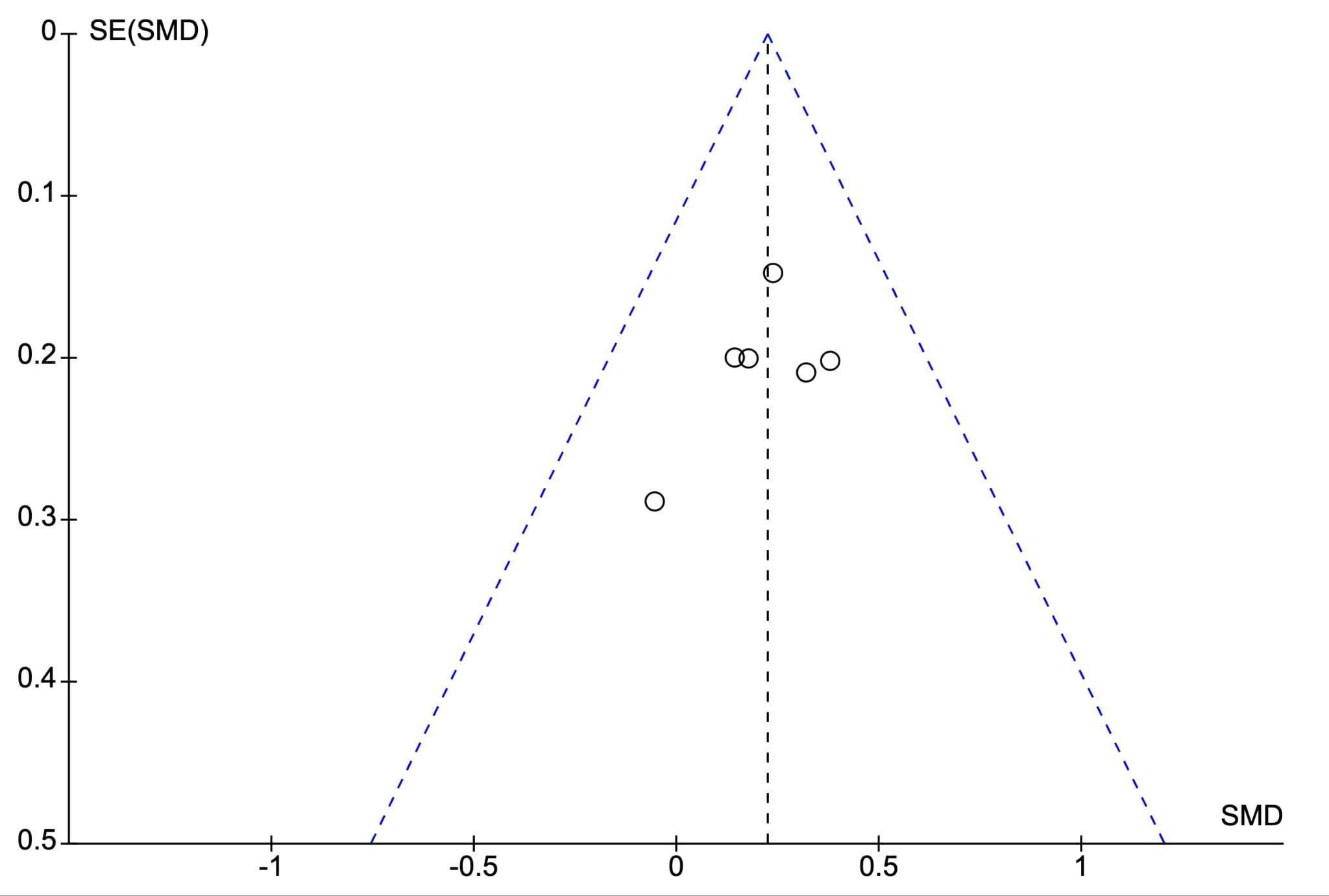


**Supplementary Figure 3. Funnel plot evaluating potential publication bias for studies reporting 12-week functional outcomes.** The 12-week funnel plot appeared broadly symmetric, with smaller studies more dispersed and larger studies clustered near the pooled effect; however, interpretation is limited by the small number of included studies.

**Forrest Plots - Exploratory Outcomes (Supplementary Material)**

**Subgroup Analysis at 12 Weeks**


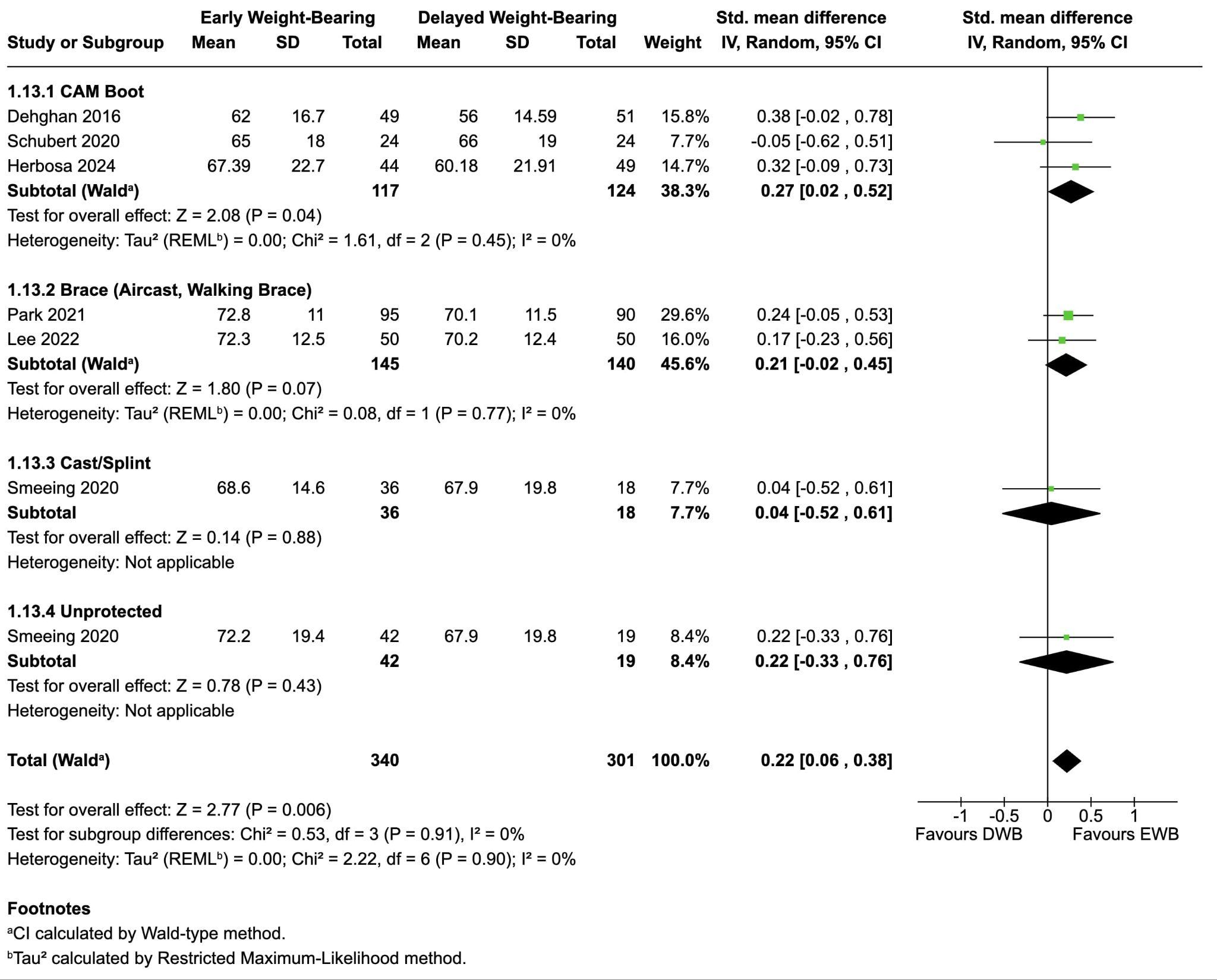


Supplementary Figure 4. Subgroup Analysis at 12 Weeks - Immobilisation Device


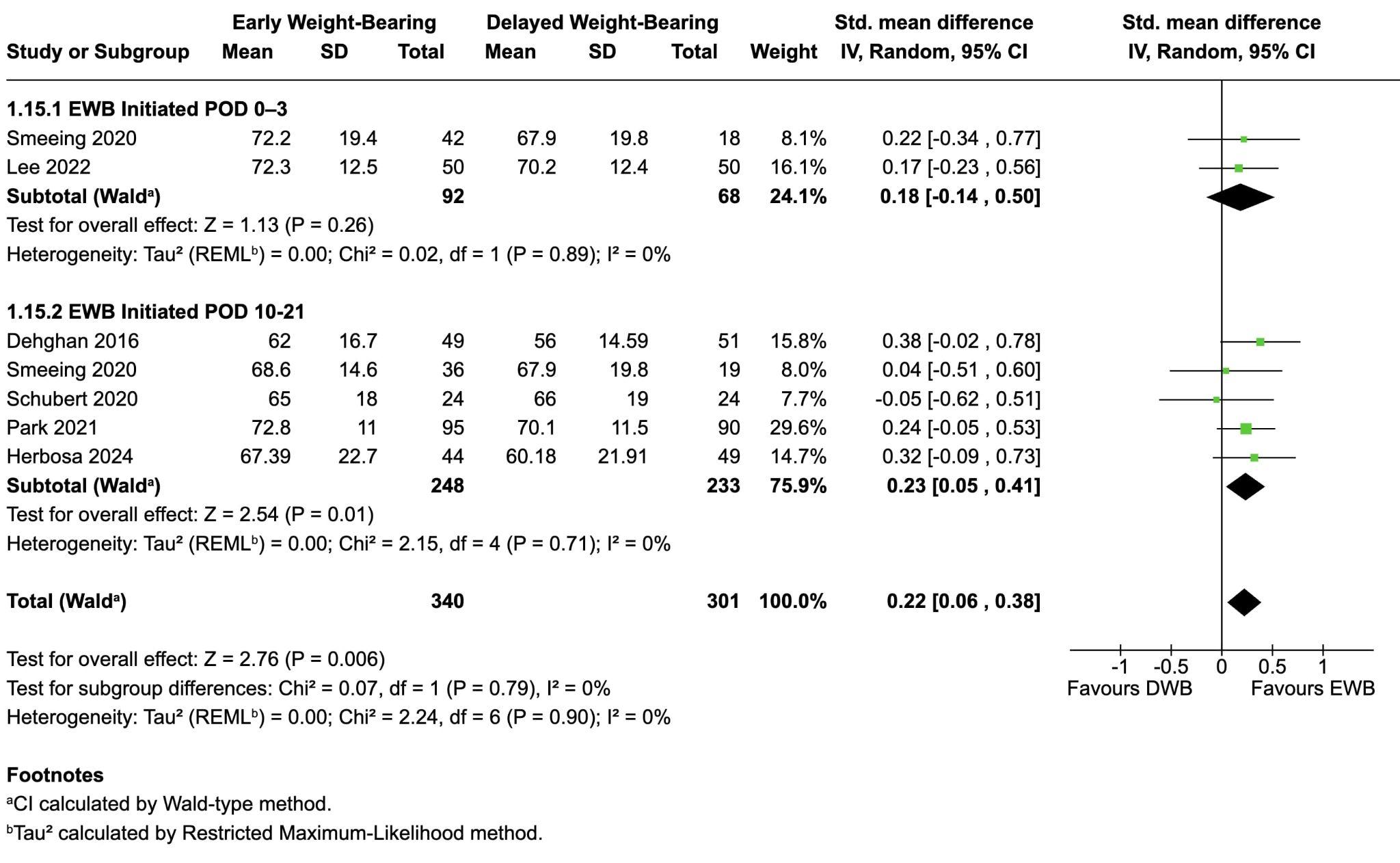


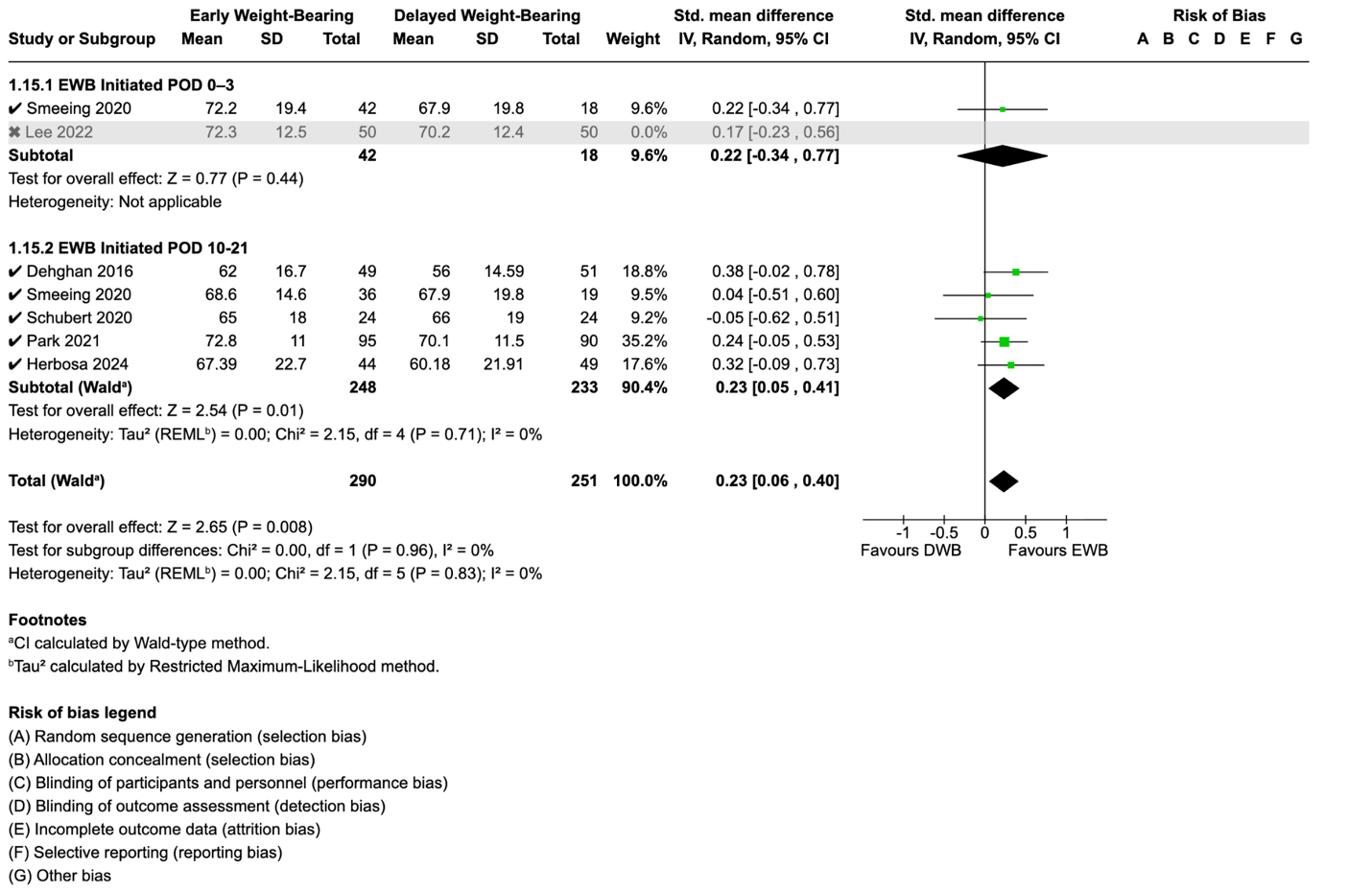


Supplementary Figure 5. Subgroup Analysis at 12 Weeks - POD Weightbearing initiation & Sensitivity Analysis

**Functional Recovery at 6 Weeks Post-Surgery**

**
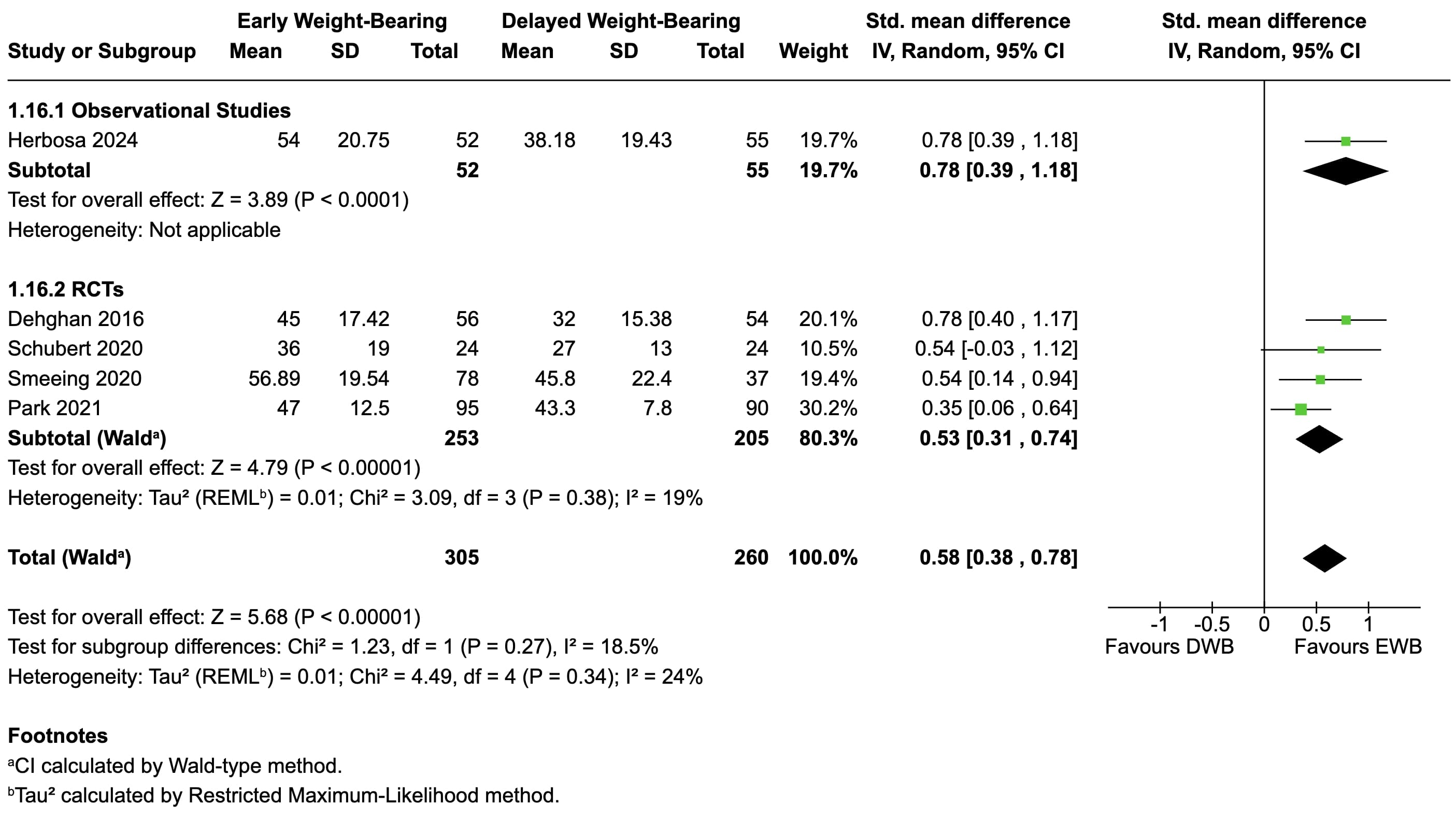
**

Supplementary Figure 6. Functional Recovery at 6 Weeks Post-Surgery

**Functional Recovery at 6 Months Post-Surgery**

**
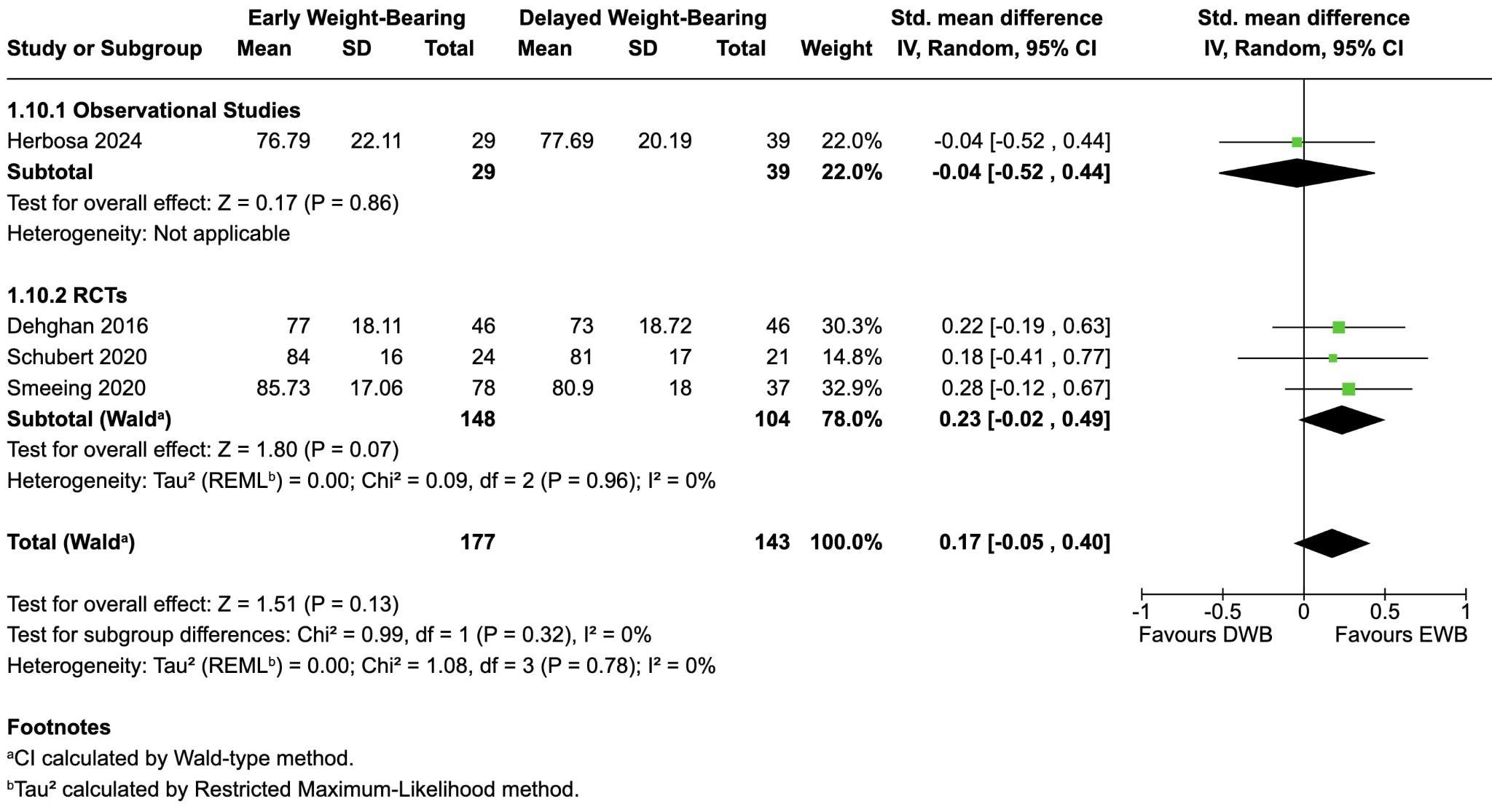
**

Supplementary Figure 7. Functional Recovery at 6 Months Post-Surgery

**Return to Work/Normal daily activities After Ankle Fracture Surgery**


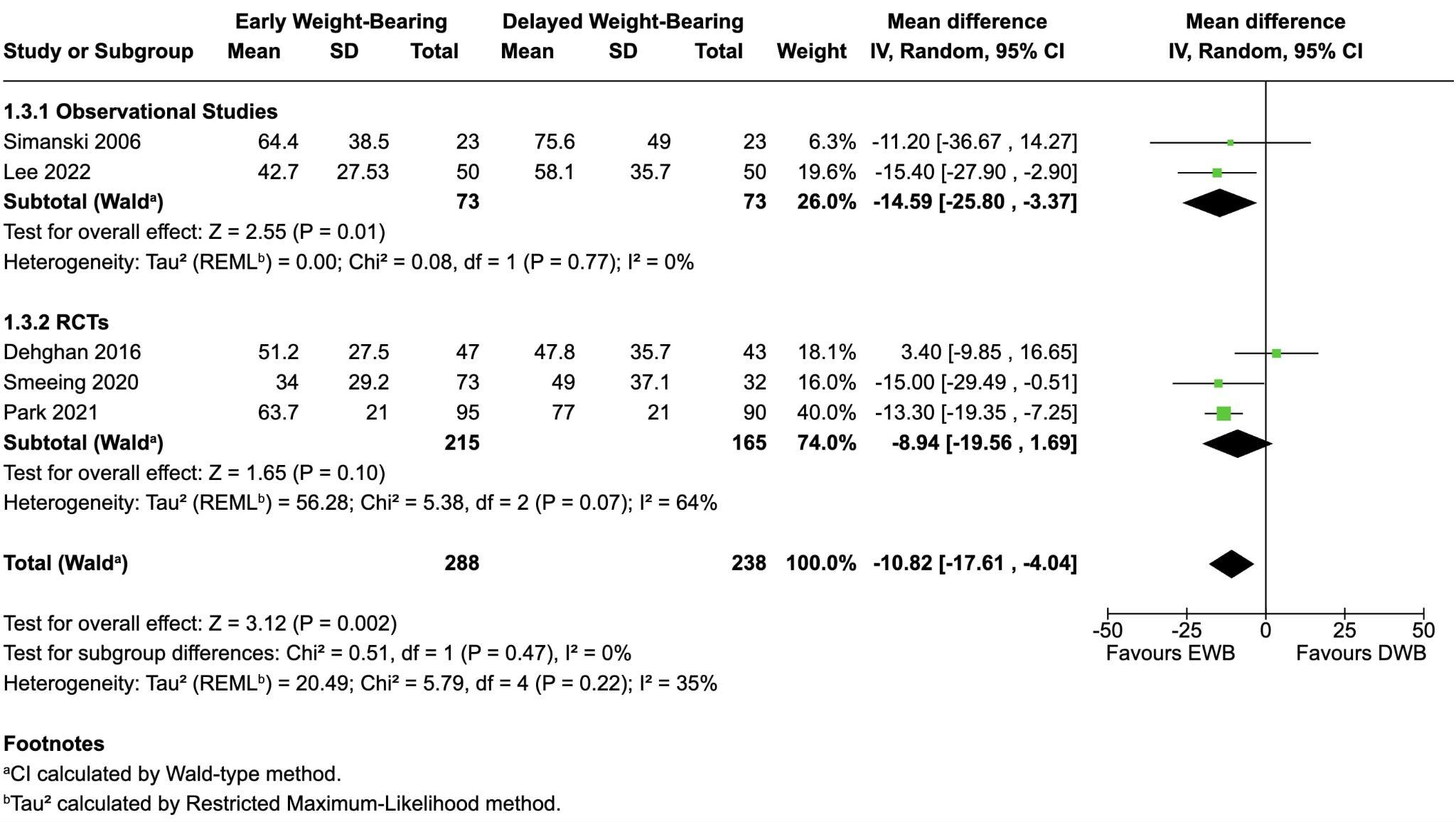


Supplementary Figure 8. Return to Work/Normal Daily Activities After Ankle Fracture Surgery

**Health-Related Quality of Life (HRQoL)**


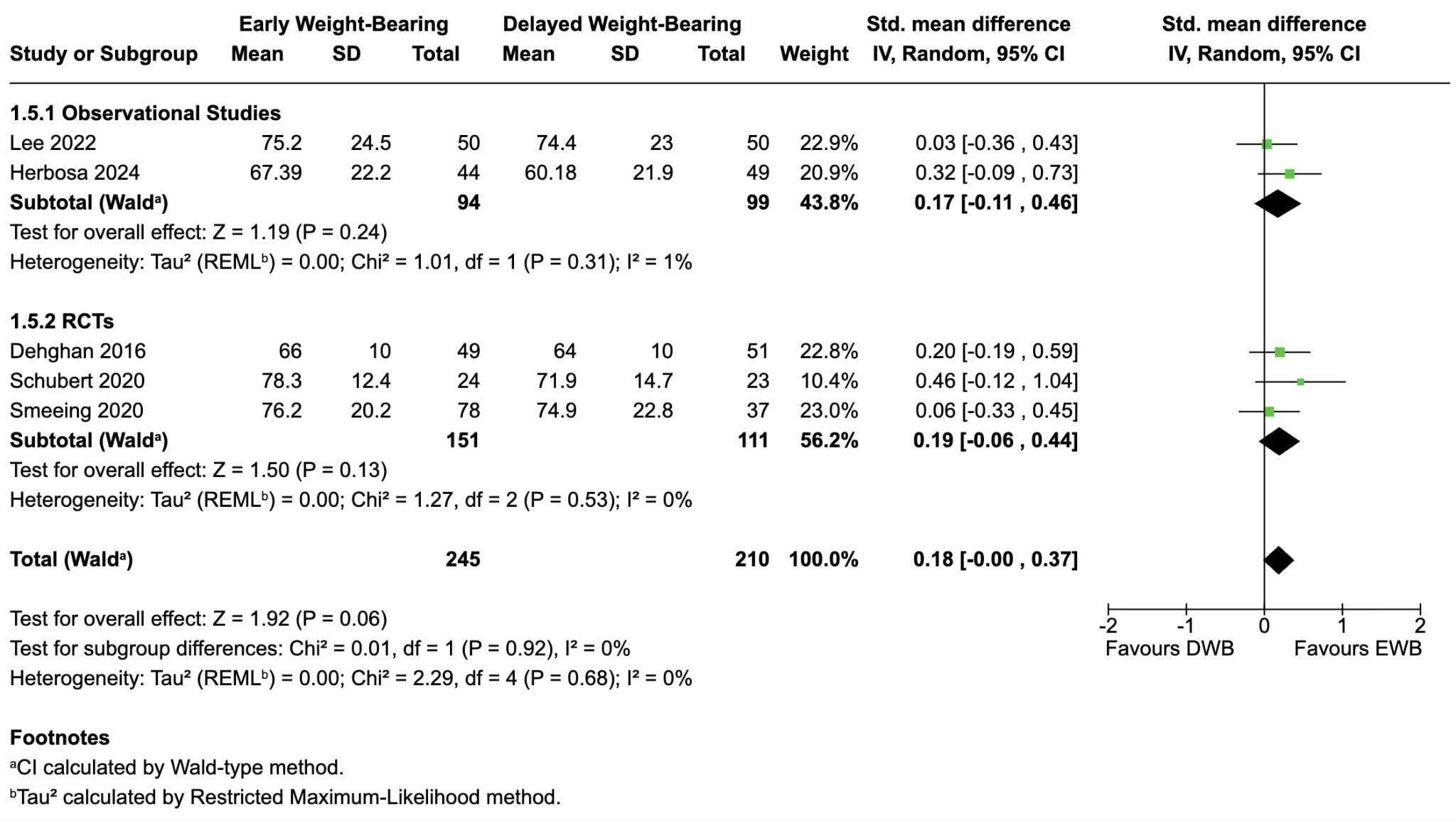


Supplementary Figure 9. Health-Related Quality of Life (HRQoL)

**
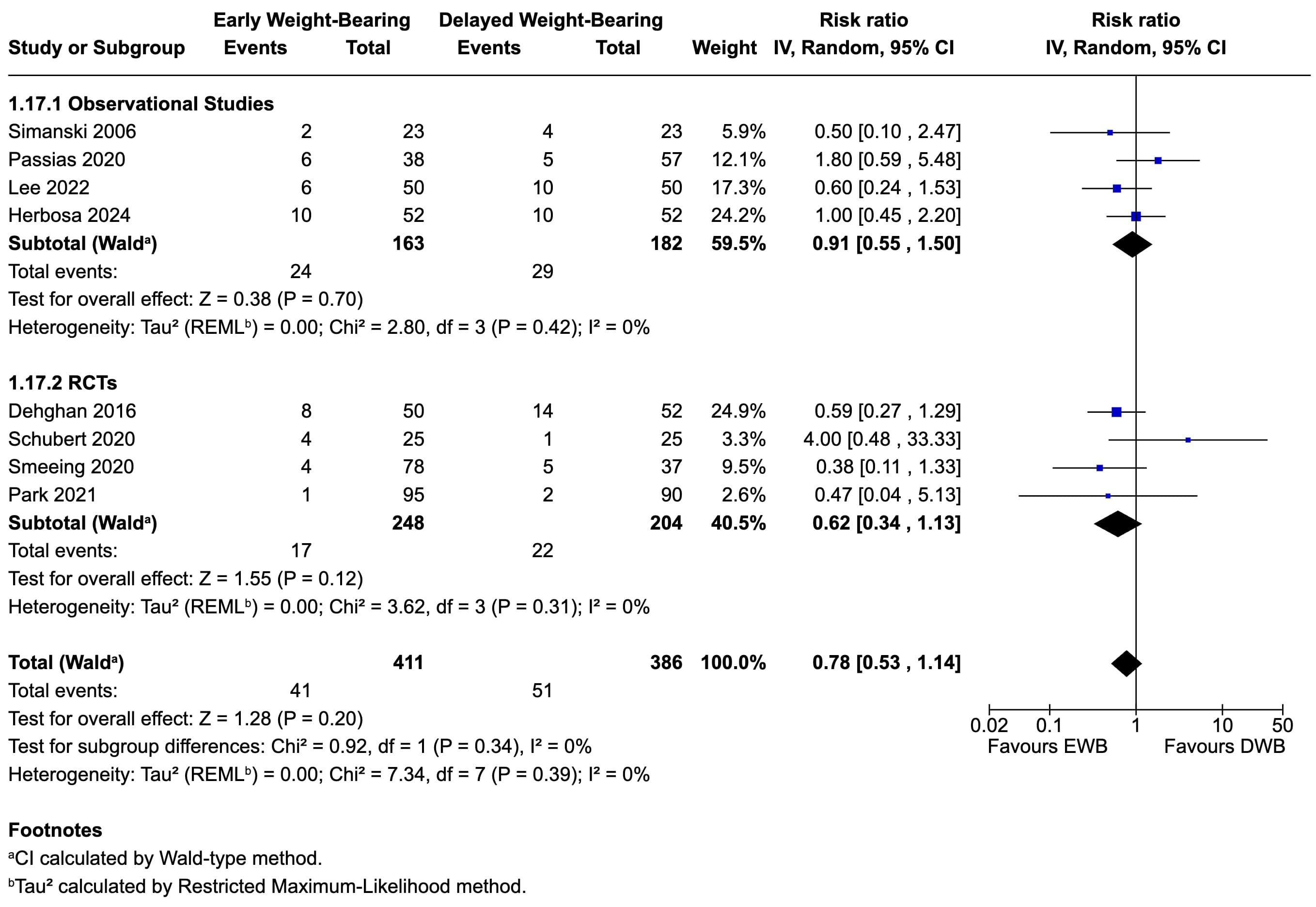
**

Supplementary Figure 10. Post-operative Complications (design stratified analysis)

Table S1. Radiographic follow-up schedules and definitions of loss of reduction in included studies (attached separately)

| Study (Author, Year) | Design | Radiographic follow-up schedule | Definition of loss of reduction (and related) | Reported events related to displacement/union |
| --- | --- | --- | --- | --- |
| Simanski, 2006 | Prospective cohort | 1, 3, 6 weeks; >12 months (AP/mortise, lateral) | NR (healing/alignment assessed by two blinded physicians) | Nonunion: 1 (DWB) |
| Dehghan, 2016 | RCT | 2, 6, 12 weeks; 3, 6, 12 months | >2 mm change vs prior films | None |
| Schubert, 2020 | RCT | 2, 6, 12, 26 weeks | Not reported | None |
| Passias, 2020 | Retrospective cohort | 3, 6, 12 weeks; 6 months; 1 year (mortise focus) | Not explicitly defined; alignment via medial clear space (normal <5 mm). Hardware loosening = lucency/back-out. Union = bridging callus at 3/4 cortices or disappearance of fracture lines; nonunion = absence of healing by 6 months | Nonunion: 2 (DWB) |
| Smeeing, 2020 | RCT | 6 weeks; 12 weeks; 6 months; 1 year | Not reported | None |
| Park, 2021 | RCT | 4, 6, 8, 12 weeks; 12 months | Loss of reduction = >2-mm displacement of the initial reduction (events counted only if not due to inappropriate activity). Implant failure = breakage or backing-out of screws/K-wires | None |
| Lee, 2022 | Retrospective matched cohort | 2 weeks; 3, 6, 12 months (AP, lateral, mortise) | >2 mm vs immediate post-op in ≥2/3 planes (AP/lateral/mortise) at 3 & 12 months; union = bridging trabeculae/osseous bone in ≥2/3 planes | None |
| Herbosa, 2024 | Prospective case–control | 2 weeks; 3, 6, 12 months | Not reported | Malunion: 1 (DWB) |

**Results and sensitivity analysis (leave-one-out method)**

Functional Recovery at 6 Weeks Post-Surgery - Sensitivity analysis confirmed heterogeneity remaining below 38% across exclusions.

Functional Recovery at 12 Weeks Post-Surgery - Sensitivity analysis confirmed heterogeneity remained at 0% across exclusions

Functional Recovery at 6 Months Post-Surgery - Sensitivity analysis was 0% across exclusions

Functional Recovery at 12 months Post-Surgery - Sensitivity analysis remained under 2% across exclusions.

Return to Work/Normal daily activities After Ankle Fracture Surgery - Sensitivity analyses demonstrated 51% heterogeneity across all exclusions.

Post-operative Complications - Sensitivity analysis confirmed heterogeneity remaining below 10% across exclusions.

HRQoL - Sensitivity analyses confirmed heterogeneity remaining below 0% across exclusions.
